# 

**Published:** 1834-04-01

**Authors:** 


3. Lancaster Lunatic Asylum .. .. .. .. .. .. .. .. .. .. .. .. .. .. 546

4. Mr. Guthrie on Stricture .. .. .. .. .. .. .. .. .. .. .. .. .. .. .. 548

5. M. Bland on the Fibrinous Polypi in the Cavities of the Heart. (Hôpital de Beaucaire) .. .. .. .. .. .. .. .. .. .. .. .. .. .. .. .. .. 551

6. Traumatic Tetanus .. .. .. .. .. .. .. .. .. .. .. .. .. .. .. .. .. 555

7. Neuralgic Affections of Stumps. (St. Bartholomew's).. .. .. .. .. .. .. 555

8. Mr. B. Cooper's Clinical Remarks on Injuries of the Head. (Guy's Hospital).. 556

9. Medical Reform .. .. .. .. .. .. .. .. .. .. .. .. .. .. .. .. .. .. 560

### Miscellanies.

FOREIGN MEDICAL POLITICS.

1. Report of the Parisian Academy of Medicine .. .. .. .. .. .. .. .. .. 561

2. Prussian System of Medical Polity .. .. .. .. .. .. .. .. .. .. .. .. 563

3. Medical Statistics and Reform.. .. .. .. .. .. .. .. .. .. .. .. .. .. 567

4. Sketch of a Plan of Practicable Medical Reform in this Country .. .. .. 569

5. Northern Provincial Meeting in Ireland.. .. .. .. .. .. .. .. .. .. .. 571

6. Obituary—Sir W. Franklin .. .. .. .. .. .. .. .. .. .. .. .. .. .. .. 572

7. Bibliographical Record .. .. .. .. .. .. .. .. .. .. .. .. .. .. .. .. 573

EXTRA-LIMITES.

1. Dr. Wise's reply to Mr. Arnott .. .. .. .. .. .. .. .. .. .. .. .. .. 577

---

### TO CORRESPONDENTS.

### PRIZE ESSAY.

The Association for Medical Reform has proposed three prizes for the best three Essays on the following subjects, viz. the Present State of Medical Science and Practice in the United Kingdom; and the most advisable and efficient Mode of promoting the Advancement and the Improvement of both, in all their Branches. The sums are 50, 30, and 20 pounds for the Essays, according to their merit. As the Essays were to be delivered on or before the 1st of March to Dr. EPPS, Great Russel Street, our notice is, most probably, too late. Still the liberality of the offers is worthy of record.

The Index to the 20 Volumes of this Series, and to the Four Volumes of a preceding Series, will now be immediately commenced.

Mr. WOOD, reprinter of this Journal in New York, is hereby informed that his letter of Feb. 27th is received. It will be complied with, in respect to both the subjects. The portrait is in hand.

HARRISON'S INFIRMARY.—Several philanthropic individuals, and especially Mr. UNDERWOOD, of Gloucester Place, have projected an Infirmary for the Relief of distorted Paupers and others needing manual and medicinal remedies. We wish it success, and have subscribed to it as a proof of our good wishes.

### BIBLIOGRAPHICAL RECORD;

FROM

*The 1st October, 1833, to 1st April, 1834.*

---

1. A Compendium of Osteology; being a Systematic Treatise on the Bones of the Human Body; designed for the Use of Students. To which is subjoined an improved Method of preparing Bones for Osteological Purposes. By Geo. Witt, M.D. Physician to the General Infirmary, Bedford. Quarto, pp. 72. Longman and Co. October, 1833. Price 7s. 6d.

☞ *See Periscope.*

---

2 Five Minutes' Advice on the Care of the Teeth. Twelfth Edition. Duodecimo, pp. 40. Renshaw and Rush, Oct. 1833.

☞ *The immense sale of this little brochure shews that it has been appreciated by the general reader, for whom it is designed.*

---

3. Traité de la Vaccine, et des Eruptions Varioleuses ou Varioliformes. Ouvrage rédigé sur la Demande du Gouvernement, precédé d'un Rapport de l'Academie Royale de Médécine. Par M. S. B. Bousquet, M.D. Octavo. pp. 367. Paris, 1833. Baillière, London.

4. A Dictionary of Practical Medicine, &c. By James Copland, M.D. Part the Second. Climacteric Decay to Dropsy. October, 1833.

☞ *We understand that upwards of four thousand copies of the first Part have been sold! Need we say more?*

---

5. An Inquiry into the Disease called Cholera Morbus, shewing its Nature, and suggesting the Means of Cure. Octavo, sewed, pp. 56. Gardiner, Princes-street. October, 1833.

---

6. Observations on Obstetric Auscultation, with an Analysis of the Evidences of Pregnancy, and an Inquiry into the Proofs of the Life and Death of the Fœtus in Utero. By Evory Kennedy, M.D. Lecturer on Midwifery, &c. Richmond Hospital, Dublin. With an Appendix, containing Legal Notes. By John Smith, Esq. Barrister at Law. Octavo, pp. 288. Dublin and London, Oct. 1833.

---

7. Illustrations of the Effects of Poisons. By Geo. Leith Roupell, M.D.

The Plates from original Drawings by AND. MELVILLE M'WHINNIE, M.R.C.S. Part I. Royal 4to, pp. 12. Four coloured Plates, price 16s. September, 1833.

---

8. An Account of the Proceedings of the first Anniversary Meeting of the Provincial Medical and Surgical Association, held at the Bristol Infirmary, July 19, 1833, containing the Address delivered on that Occasion by ED. BARLOW, M.D. Physician to the Bath United Hospital, &c. Octavo, pp. 71. 1833.

---

9. CYCLOPÆDIA OF PRACTICAL MEDICINE, Part XVIII. Pregnancy to Roseola. Oct. 1833.

☞ *In this Part, there are some very clever articles by* Dr. TODD, Dr. CUMIN, Dr. HALL, Dr. TWEEDIE, Dr. THOMSON, Dr. BARLOW, *and* Dr. BEATTY.

---

10. Syllabus of a Course of Lectures on the Principles and Practice of Surgery. By FRED. TYRRELL, Surgeon to St. Thomas's Hospital, Octavo, pp. 116. 1833.

☞ *If the* bodies *of these Lectures be proportionate to the* heads, *we venture to say they will be very valuable to the student.*

---

11. Mental Culture; or the Means of developing the Human Mind. By J. L. LEVISON. 8vo, pp. 300. London, 1833.

☞ *This little Work contains an immense fund of important information on the subject of cultivating the mind, by improving the various faculties. It is based on phrenology, and the matter is too elementary to be susceptible of analysis in a medical journal.*

---

12. A new Method of making Anatomical Preparations; particularly those relating to the Nervous System. By JOSEPH SWAN. Third Edition, considerably enlarged, pp. 111. Longman, and Co. 1833.

☞ *This Edition should be in the hands of all those who are in the habit of making anatomical preparations.*

---

13. The Principles and Practice of Obstetric Medicine, &c. By DAVID D. DAVIS, M.D. Professor of Midwifery, &c. Parts XXIV. XXV. and XXVI. with Plates. October, November, and December, 1833.

---

14. The Register and Library of Medical and Chirurgical Science—a Medical Newspaper, edited by GRANVILLE SHARPE PATTISON, M.D. Professor of Anatomy in Jefferson College, Philadelphia. No. I. pp. 64. Washington, 1833.

☞ *This is a bold as well as novel attempt of our talented countryman. In one single sheet, which folds as a newspaper, are contained* 64 *pages of small type—mostly in double columns. Dr. Pattison proposes to re-publish all the best medical works in this cheap form, besides the varieties of a medical journal. Mr. Bell on the Nerves is re-printed in the first Number. It is to come out weekly. This is a Herculean task; but we think it will succeed, and form a kind of epoch in periodical literature. We need hardly say, that we wish Dr. Pattison even more success than he himself anticipates.*

---

15. A Memoir on the Advantages and Practicability of dividing the Stricture in Strangulated Hernia on the Outside of the Sac, with Cases and Drawings. By C. ASTON KEY, Senior Surgeon to Guy's Hospital, &c. Octavo, pp. 161. London, 1833.

---

16. Surgical Observations on the Restoration of the Nose, and on the Removal of Polypi and other Tumours from the Nostrils:—from the German of Dr. Diffenbach, of Berlin; with the History and Physiology of Rhinoplastic Operations, Notes and additional Cases. By JOHN STEVENSON BUSHNAN, M.R.C.S. &c. Surgeon to the Dumfries Public Dispensary. Octavo, pp. 160, and 26 Plates, price 12s. Highley, Oct. 1833.

---

17. Thoughts on Medical Reform. By a Retired Practitioner. Octavo, pp. 33, sewed. Oct. 1833.

---

18. Fasciculi 1, 2, 3, 4, price Two Shillings each. A Series of Anatomical Plates, in Lithography, with References and Physiological Comments, illustrating the Structure of the different Parts of the Human Body. Edited by JONES QUAIN, M.D. Professor of Anatomy in the University of London. Division I., the Muscles. Folio. Taylor, Oct. 1833.

☞ *This is one of the cheapest systems of anatomical plates that we have seen: Dr. Quain's name is a sufficient guarantee for the correct execution of the Work.*

---

19. Chemical Diagrams, accompanied with a concise Description of each Decomposition. The vegetable alkalies, the Urine and Urinary Calculi, &c. intended to facilitate the Progress of the

Medical Student. By Alex. Lee, Surg. Editor and Translator of Celsus. Small 8vo, pp. 182. Cox, Borough, 1833, price 7s.

☞ *This is a very useful little volume for ready reference.*

20. Surgical Essays, the Result of Clinical Observations made at Guy's Hospital. By Bransby Cooper, Esq. F.R.S. &c. Large 8vo, pp. 281. London, 1833.

21. First Annual Report of the Sussex and Brighton Infirmary for Diseases of the Eye, established August, 1832.

22. A Practical Treatise on Diseases of the Joints. By W. J. Wickham, Surgeon to the County Hospital, Winchester. Octavo, pp. 177, two Plates. Highley, 1833.

23. Clinical Observations on the Constitutional Origin of the various Forms of Porrigo, &c. with Directions for the more Scientific and Successful Management of this obstinate Class of Diseases, &c. By Geo. Macilwain, Surgeon to the Finsbury Dispensary, &c. Octavo, pp. 83. Longman and Co. 1833.

24. Relacao Historica, Statistica e Medica da Cholera Morbus em Paris, precedada da Topographia desta Capital. Por Francisco d'Assis Soaza Vaz, Doutor em Medicina, &c. Octavo, pp. 372. Paris, 1833.

☞ *Dr. V. appears to have carefully observed the epidemic in Paris; and, as the disease is now scourging his own country (Portugal), his Work will prove exceedingly useful there. In this country, cholera has, for the present, ceased to excite either interest or apprehension.*

25. The History of a Case, in which Animals were found in Blood drawn from the Veins of a Boy, with Remarks. By J. Stevenson Bushnan, F.L.S. Surgeon to the Dumfries Dispensary, &c. Octavo, pp. 74, with a coloured Plate. Highley, price 3s. 6d.

26. Obstetric Tables, comprising coloured Delineations on a peculiar Plan, intended to illustrate elementary and other Works on the Practice of Midwifery, elucidating particularly the Application of the Forceps and other important Practical Points in Obstetric Science. By G. Spratt, Surgeon-Accoucheur, Editor of the Flora Medica, &c. Quarto, with Twelve Plates, coloured.

☞ *This Work is very ingeniously contrived, and will be found of the greatest advantage to the student of midwifery.*

27. Introduction to the Study and Practice of Midwifery, and the Diseases of Women and Children. By Wm. Campbell, M.D. late Surg. R.N. Lecturer on Pathology and Practice of Medicine, Midwifery, &c. Octavo, pp, 714. Edinb. 1833.

☞ *This appears to be a very laborious and careful compilation, imbued with a more than ordinary proportion of original observation. It is well calculated as a class book for Students in midwifery —especially those who are attending the author's lectures.*

28. A rational Exposition of the Physical Signs of the Diseases of the Lungs and Pleura, illustrating their Pathology and facilitating their Diagnosis. By Ch. J. B. Williams, M.D. Second Edition. Churchill, Dec. 1833

29. On the Efficacy of the Secale Cornutum in Hæmorrhage and Leucorrhœa, and on its Effects in Gonorrhœa. By E. Negri, M.D. Read before the Medical Society of London, 25th Nov. 1833.

☞ *See Periscope.*

30. A Literal Interlinear Translation of the First and Third Books of Celsus; with Ordo, and the Text of Targa. By Charles Gerard. Revised and amended, with an Introduction, &c. by Robert Venables, A.M. M.B. Octavo, pp. 250. Ed. Portwine, Dec. 1833.

31. The Nature and Treatment of the Epidemic Cholera; with Instructions for the Suppression and Prevention of the Disease. By Robert Venables, A.M. &c. Octavo, pp. 52. Second Edition. Ed. Portwine, Dec. 1833.

32. A Treatise on the Diseases of Females. By William Dewees, M.D. of Philadelphia. One vol. 8vo. pp. 591. 4th edit. greatly improved. 1833.

33. On the Penetration of Gases. By J. K. Mitchell, M.D. Professor of Chemistry, &c. From the American Journal of Medical Science.

34. Beobachtungen Urspruenglicher und Gaenzlichen Mangels der Augen Bei Menschen und Thieren, Von D. Burkhard Wilhelm Seiller. Mit Einer Rupfer—und einer Steindruckt a fel. Quarto. Dresden, 1833.

35. A Series of Anatomical Plates, &c. &c. By Jones Quain, M.D. Fasciculus V. The Muscles continued.

☞ *These plates keep up their character.*

36. A Demonstration of the Nerves of the Human Body; consisting of Four Parts. I. The Cervical and Thoracic Portions of the Sympathetic, and the Nervesof the Thoracic Viscera. II. The Lumbar and Sacral portions of the Sympathetic and the Nerves of the Abdominal Viscera. III. The Cerebral Nerves. IV. The Spinal Nerves. Part IV. Price Four Guineas. Longman and Co. 1834.

☞ *This work is beyond all praise.*

37. The Dispensatory of the United States of America. By Geo. B. Wood, M.D. and Franklin Bache, M.D. 8vo. pp. 1073. Philadelphia, 1833.

☞ *This appears to be a highly valuable compilation, embracing much that is new in this country.*

38. An Essay on the Physiology of the Iris; with a different View of its Relations and Sympathies from the one usually received; and attempt to establish a new Theory of the Action of Light, &c. By John Walker, Assistant Surgeon to the Manchester Eye Institution, &c. Octavo, pp. 16. 1833.

39. An Examination into the Causes of the declining Reputation of the Medical Faculty of the University of Edinburgh, and the Origin of another class of Medical Professors, commonly called "Private Lecturers," &c. 8vo, pp. 58, Edinburgh, 1834.

☞ *This is of local interest, and shews no extended views of medical education. On the contrary, the Pamphleteer thinks that too many classes are included in the Edinburgh curriculum, and too much study enjoined!!*

40. Illustrations of all the most celebrated Medical and Surgical Works, comprising a complete System of Morbid and Descriptive Anatomy, wherein is combined, with variety in each number, methodical arrangement of the whole. Nos. 1 and 2. Six plates weekly, price Threepence each. Dulau and Co. Soho Square. 1834.

☞ *Of all the cheap works which we have yet seen, this is the cheapest. We think it is hardly possible that such plates and letter-press, at threepence per plate, can be continued, without ruin to the publishers. It is a most extraordinary publication!!*

41. Elements of Materia Medica and Therapeutics; including the recent Discoveries and Analyses of Medicines. By A. T. Thomson, M.D. F.L.S. &c. Vol. II. pp. 694. 1834. Longman and Co.

42. The Monthly Journal of Medico-Chirurgical Knowledge. Published by H. Gouraud, A. Trousseau, J. Lebrudy. Translated by Henry Belfield Lefevre. 1st Fasciculus, Oct. 1, 1833. Price 1s.

☞ *We wish this attempt of our continental brethren every success. But we have some fears for the result.*

43. I. Statement of the New Regulations published by the University of St. Andrew's.

II. Protest of the Examinators to the University of St. Andrew's, against the Petition of the Royal College of Surgeons of Edinburgh, &c.

☞ *At a time when the subject of general reform in the medical profession is agitated, we cannot enter into the quarrels of particular universities or colleges.*

44. A Treatise on Diseases and Injuries of the Nerves. By Joseph Swan. a new edition, very considerably enlarged. Octavo, pp. 356. Ten Plates. Price 14s. boards. Longman and Co. 1834.

☞ *To be reviewed.*

45. Thoughts on Materialism and on Religious Festivals and Sabbaths. By Henry Bradshaw Fearon. 8vo, pp. 214. Longman and Co. 1834.

46. An Investigation into the remarkable Medicinal Effects resulting from the external Application of Veratria. By Alex. Turnbull, M.D. Octavo, pp. 96. Longman and Co. 1834.

47. Principles and Practice of Obstetric Medicine, &c. By D. D. Davis, M.D. Parts XXVII. and XXVIII.

48. A Series of Anatomical Plates in Lithography, with References and Physiological Comments, &c. Edited by JONES QUAIN, M.D. Division 1, Muscles. Fasciculi VII. and VIII. January and February, 1834.

---

49. A Treatise on the Circulation of the Blood, in two Parts. Part I. containing an Explication of the Anomalies, res inepta, &c. of the present Doctrine. Part II. an Attempt to explain how the Circulation is accomplished by motive powers different to those which are supposed to effect that Operation. By J. F. HANDLEY. Octavo, pp. 33. 1834.

---

50. The Anatomy and Surgery of Inguinal and Femoral Hernia. Illustrated by Plates coloured from Nature, and interspersed with Surgical Remarks. By E. W. TUSON. F L.S. Assistant Surgeon to the Middlesex Hospital, Lecturer on Anatomy, &c. Folio, Churchill, 1834.

---

51. The Principles of Diagnosis. Vol I. Second Edition. By MARSHALL HALL, M.D. F.R.S. L. & E. Octavo, pp. 426. January 1834.

☞ *This Edition appears to be much improved, and, as the author remarks, "that the matter of the second volume is of much more practical interest than that of the present one," we shall look with anxiety for its appearance, and we will not fail to give an early account of it in this Journal.*

---

52. On the Influence of minute Doses of Mercury, combined with the appropriate Treatment of various Diseases, in restoring the Functions of Health, and the Principles on which it depends. By A. P. W. PHILIP, M.D. F.R.S. &c. Duodecimo, pp. 112. London, Renshaw, 1834.

☞ *The substance of this little volume was published in the Medical Gazette, some years ago. The pathological portions are much enlarged in this compendious re-publication.*

---

53. Lectures on the Morbid Anatomy, Nature, and Treatment of Acute and Chronic Diseases; delivered in the Theatre of Anatomy, Webb Street. By the late JOHN ARMSTRONG, M.D. Edited by JOSEPH RIX, Member of the Royal College of Surgeons. Octavo, pp. 851. Price 16s. London, Baldwin and Co. 1834.

☞ *This, from the testimony of Dr. A. himself, is the most correct edition of his valuable lectures that has ever appeared.*

---

54. The second Fasciculus of Anatomical Drawings, selected from the Collection of Morbid Anatomy in the Army Medical Museum at Chatham. Folio, nine plates, with descriptive Letter-press. 1834.——*To be noticed in our next.*

---

55. The Anatomy and Physiology of the Liver. By FRANCIS KIERNAN, Esq. Member of the R.C.S. late Teacher of Anatomy. (From the Philosophical Transactions.)

---

56. On the Reflex Function of the Medulla Oblongata and Med. Spinalis. By MARSHALL HALL, M.D. (From the Philosophical Transactions.) *Under Review.*

---

57. Of Monopolies in Learning; with Remarks on the present State of Medical Education, and on the Constitution of the Scotch Universities. By ANDREW BUCHANAN, Graduate and Regent of the Faculty of Medicine in the University of Glasgow. 8vo, pp. 24. Glasgow, 1834.

☞ *This pamphlet is deserving of perusal at the present moment.*

---

58. Observations on the Ulcerative Process and its Treatment, particularly when affecting the Leg. By WILLIAM ECCLES, Surgeon. 8vo, pp. 66. Highley, 1834.

---

59. A Treatise on Lesser Surgery; or the minor Surgical Operations. By M. BOURGERY, M.D. &c. Translated from the French, (with Notes and an Appendix.) By W. C. ROBERTS and JAMES B. KISSAM. New York, 1834, pp. 376, and Appendix, pp. 36.

☞ *We do not believe that there is a volume of the same size in the English, or in any other language, which contains such a magazine of truly useful, because practical information, as that which Mess. Roberts and Kissam have translated, and enriched by notes and appendix. It will prove a treasure to the student and young practitioner, while it will serve as a most valuable reference even to the more experienced surgeon.*

### LITERARY NOTICE.

*Preparing for the Press,*—The entire Works of JOHN HUNTER, F.R.S. with Notes by J. F. PALMER, Senior Surgeon to the St. George's and St. James's Dispensary ; assisted by G. G. BABINGTON, Surgeon to St. George's Hospital, Lecturer on Surgery at St. George's Hospital, formerly Surgeon to the Lock Hospital; THOMAS BELL, F.R.S. F.L.S. Lecturer on the Teeth, and Lecturer on Comparative Anatomy at St. George's Hospital; ROBERT LEE, M.D. F.R.S. Physician to the British Lying-in Hospital, Physician to the St. Marylebone Infirmary, and Lecturer on Midwifery; and RICHARD OWEN, M.R.C.S. F.Z.S. Assistant Conservator of the Hunterian Museum.

We can scarcely doubt but this announcement of a complete, and, at the same time, cheap edition of Mr. Hunter's invaluable writings, will be regarded with general satisfaction by the whole profession. The names of the Editors, and the appropriation of the several parts of the work, are, in our opinion, most judicious, and warrant a full expectation, that the notes will be ably executed, and those additions made which the progress of science has rendered necessary. We understand that the Royal Society have, with great liberality, granted the use of the whole of their copper-plate engravings connected with Mr. Hunter's papers in the Philosophical Transactions, for the purpose of enabling the Editor to complete the series of plates designed for the illustration of this edition. We are informed that the Editor has made great exertions to collect the most authentic materials for a new Life of the Author, and that it is his intention to publish a collated copy of his MS. Surgical Lectures.

*Just published, price 7s. 6d.*

THE RECESS; or, Autumnal Relaxation in the HIGHLANDS and LOWLANDS—being the Home Circuit *versus* Foreign Travel; a Tour of Health and Pleasure to the Hebrides. By JAMES JOHNSON, M.D.

" The author of this book, with a right proper feeling of love for the mother-land, prefers the Highlands and Lowlands of Scotland to the romantic scenery breasting the Garonne—in short, to the whole of the Continent. His book is full of this feeling. He treats his subjects with freshness and earnestness, and evidently has his heart in what he is doing. He is so engrossed in the *locale*, that he succeeds in creating a strong interest in the reader. His book will prove a lively companion on the route it traces."—*Atlas.*

" The Author, who has evidently a turn for the satirical, seems to have had abundant materials afforded him for the gratification of his humour. The districts through which he travelled abound in romantic scenery, and of a character to compensate highly those who travel for amusement or health. A better companion than this book they can hardly find."—*News, Feb. 9th*, 1834.

" It is but justice to the Author to say that he is as pleasant a companion as we have met with for a long time. No person need desire a more agreeable way of spending part of a winter's night or of a summer's day than to accompany him in his tour through Scotland and back again. He is evidently a person who has had frequent and familiar intercourse with lake and mountain in all parts of the world. He enjoys nature in every feature, whether of beauty or terror, and communicates his feelings with great vivacity and power of description. It is very clear that he thought much upon various subjects, and, though he makes no parade of his acquirements, they now and again slip into his narrative in a way which shows that he could, if he pleased, sport a pamphlet on the poor laws or the corn trade as well as other people. The author is no vulgar every-day traveller, as will be very soon apparent to those who look into his tour."—*Morning Post, 22d February*, 1834.

" HERE'S food for reflection!—a banquet of mind,
Worth half the old books in the 'BRERA!'
A feast for the Attican palate, combined
With a spice from the wit of 'ABDERA!' "*

*From the Author of the* HELIOTROPE.

" The " list of contents" which commences this volume made us, on a first glance, feel half inclined to close it again; but, still reluctant, as we always are, to let any task, however Herculean, conquer us, we commenced the first two or three pages, and the further we followed the tourist, the more disinclined we were to leave a line unread; and, we must confess, we know not when we have been better pleased. The object of this volume is to prove to our English tourists, who annually flock to foreign shores, that our own islands present subjects of equal, if not greater, interest to the contemplative traveller, than may be found in any parts of the Continent.

Where so many lively descriptions, blended with such judicious and moral reflection abound, it were difficult to select any particular passage for commendation; therefore, we cannot do better than to recommend the volume to our readers." *Satirist, Feb. 8th*, 1834.

" A very clever and entertaining volume. To the concluding word '*vale*,' we are sure the response will be, *not* vale; but a desire speedily to travel with the same agreeable and entertaining companion again. It is but justice to the author to say that he is generally lively; and, when in the heart of the Highlands, descriptive and pleasant. He has also given us some poetical varieties, which do not deteriorate from his prose abilities."—*Literary Gazette, Feb. 15th*, 1834.

He gives us, nevertheless, some pleasing descriptions—nay, passages in which manners are cleverly delineated—and has such good will towards the land, that he often speaks the truth about it. This traveller is the kindest of all tourists: he seeks to extract enjoyment out of every thing, and he goes smiling over the land, scattering his jokes and his jibes like a prodigal."—*Athenæum*, 1*st March*, 1834.

" In this trial of skill, we give judgment for the *plaintiff*, and condemn the *defendant*, Foreign Travel, to all the costs of the journey. We have seldom read a more lively, pleasant, rattling tour. We wish that all travellers would act and write as he has done. We should then alter the proverb a little; and, instead of saying—" that travellers see strange things," it would stand thus—" they say good ones." If any one wishes to employ an hour both rationally and pleasantly, we say it with reflection, and not as mere words of course, they cannot do better than bestow that hour upon this book. We were really both amused and surprized, when we perused it."—*Metropolitan Magazine, March 1st*, 1834.

* Democritus.

### INDEX.

A.

Abattement, explanation of ........ 98
Abdomen, wounds of, B. Cooper on .. 139
Abscess, pulmonary, obscure case of, 192
Abscess of the Liver, Mr. Geddes on .. 365
Abscess of the mamma, Dr. Beatty on 448
Academy of Sciences, proceedings of, 231
Academy of Medicine, proceedings of, 235
Acetas plumbi in pulmonary diseases, 193
Acid, nitric, effects of a large dose of 64
Admiralty Office, new regulations from 188
Adulterations of fecula ............ 516
Affections, calculous, statistics of.... 233
Ague treated by salicine .......... 482
Air, Dr. Graves on the secretion of .. 442
Aldersgate question .............. 185
Amenorrhœa, causes of............ 161
Amenorrhœa, cured by sinapisms to the mammæ .................. 171
America, cholera in .............. 179
Amputation in a seven weeks' infant, 191
Amputation of the leg by a ligature.. 235
Amputation in spreading gangrene .. 265
Anatomical plates recently published.. 332
Anatomical plates, Mr. Swan's...... 334
Anatomical plates, Mr. Quain's .... 335
Anatomical drawings in the Museum at Chatham...................... 304
Anatomy, morbid, Dr. Hope on .... 66
Anatomy, morbid, Dr. Carswell on .. 66
Anatomy, morbid, Cruveilhier on ... 66
Anatomy of the liver, Mr. Kiernan's.. 305
Anatomy of the eye, Dr. Arnold on the 490
Aneurism, internal, cases of ........ 261
Aneurism of the aorta, cases of .... 262
Aneurisms, anastomosing, treatment of 219
Animalculæ in living blood ........ 39
Anne of Austria, sterile for 21 years, 159
Anterior membrane of the eyeball .. 446
Antimony, use of, in cholera........ 145
Antiphlogistic treatment of gonorrhœa 276
Apoplectiform disease, curious case of 164
Apoplexy, Dr. Uwins on .......... 57
Armies, mortality in .............. 231
Arnott, Mr. case of œsophagotomy .. 172
Arsenic, effects of a large dose of.... 63
Artery, subclavian, ligature of ...... 206
Artery, gluteal, ligature of the ...... 165
Ascherson, M. on congenital fistula of the neck ...................... 481
Ascites cured by tapping, case of .... 543
Ascites, curious case of............ 177
Asphyxia, case of, during intoxication 196
Astragalus, dislocations of the ...... 520
Atkinson, Mr. his medical bibliography 329
Audible signs of pregnancy ........ 100
Auscultation, obstetric, Dr. Kennedy on .......................... 92
Auscultation, mediate, M. Piorry on.. 337
Auscultation, application of, to still-born children .................. 164

B.

Ballottement, explanation of........ 99
Bangalore, climate of.... ........ 362
Baregine, description of............ 232
Barry, Sir David, his factory report.. 393
Beatty, Dr. on uterine hæmorrhage.. 446
Bell, Sir C., doctrine of the nerves confirmed ...................... 225
Bell, Sir C., letter to the Editor .... 228
Berlin, report of hospital at ........ 218
Bibliography, medical, Mr. Atkinson's 329
Bladder, the removal of clots in the.. 183
Bleeding, Mr. Wardrop on.......... 476
Bleeding, injurious effects of........ 476
Blood, animalculæ found in the...... 39
Blood, alterations of, in disease...... 381
Blood, Muller's experiments on the .. 501
Boismont, M. on the plica polonica .. 215
Bones, mode of cleansing .......... 150
Bony union of fractured cervix femoris 170
Bougies, use of, in amenorrhœa .... 161
Bouillaud, M. on typhus fever ...... 514
Bouillaud, M. on typhus fever ...... 524
Bousquet, M. on vaccination ...... 417
Bowels, fatty discharge from the .... 8
Bower, Mr. his clinical report ...... 561
Brain, asthenic disease of, Dr. Uwins on 57
Bright, Dr. on diseases of the pancreas 1
Bright, Dr. on diseases of duodenum, 1
Bristol, medical school of .......... 181
Bristol Medical Conversation Society, 188
Bronchus, tooth lodged in the ...... 471
Brookes, Joshua, portrait of . ...... 189
Bruit, placentary, Dr. Kennedy on .. 101
Burn, cicatrix from a, removal of.... 249
Bushnan, Mr. on animalculæ in blood, 39

C.

Cadmium, sulphate of, for corneal specks ........................ 485
Cæsarian operation, case of ........ 207
Cæsarian operation, case of ........ 221
Calamine powder, use of, in small-pox, 60
Calcareous incrustation on the lens .. 501
Calculous affections, statistics of .... 233
Calcutta, transactions of the Medical Society of ...................... 361
Cancer of the heart and kidney...... 236
Carcinoma, varieties of ............ 69
Carcinoma, chemical characters of .. 71
Carlile, Mr. on sounds of the heart .. 134
Carmichael, Mr. his reclamation .... 285

Carmichael, Mr. ligature of the gluteal artery ........ 165
Carswell, Dr. on carcinoma ........ 68
Carswell, Dr. on pathological anatomy 289
Cath. de Medicis, sterile for 10 years.. 159
Cave, vapour, at Pyrmont.......... 201
Cephalæmatomia, description of .... 210
Cerebral diseases, Dr. Paine on...... 433
Cerebral affection, complicated with jaundice .................. 479
Cervix femoris fractured, bony union, 170
Cherry-laurel water for neuralgia.... 480
Cholera, state of intestinal glands in, 67
Cholera, Cruveilhier on the ........ 74
Cholera, state of alimentary tube in.. 76
Cholera, Asiatic, Mr. Langford on .. 145
Cholera in Philadelphia ............ 178
Cholera, Dr. Tyler's doctrine of .... 180
Cholera at New Orleans............ 239
Cholera, treatment of.............. 505
Choleraphobia in Scotland.......... 456
Chromate of potass, properties of.... 507
Chronic diseases, use of whey in .... 197
Cicatrix from a burn, removal of.... 249
Climate, effects of, on fecundity .... 162
Clinical lectures of Dr. Stokes ...... 257
Clinique of M. Dupuytren........... 241
Clinique of M. Ricord ............ 245
Clinique of M. Bouillaud .......... 254
Colon, abnormal flexure of ........ 200
Conception, without consciousness .. 131
Condylomata, venereal, lotion for.... 204
Cooper, B. surgical essays.......... 137
Cooper, B. on wounds of the abdomen 139
Cooper, B. on rupture of the jejunum, 142
Cooper, B. on rupture of the spleen.. 144
Cooper, Mr. B. on injuries of the head 556
Copland, Dr. his Dictionary of Practical Medicine .................. 46
Copland, Dr. on diarrhœa.......... 47
Corpora lutea, their anatomical characters...................... 128
Corpora lutea, produced by impregnation ...................... 129
Corpora lutea, Dr. Montgomery on .. 127
Corrigan, Dr. on sounds of the heart, 134
Corruption in medical charities ..... 186
Cow-pox, origin of................ 504
Cow-pox, artificial production of .... 208
Craniotomy, child lived after ....... 204
Creosote, its medicinal properties.... 497
Croton oil, internal use of.......... 255
Croup, use of tartar-emetic in ...... 212
Cruveilhier's Anatomie Pathologique 303
Cunningham, Mr. on retroversio uteri 156
Cures, spontaneous, of dropsical ovaria 206
Cynanche, laryngea, Mr. Robertson on 155

D.

Davids, Mr. case of wounded intestine 182
Davis, Dr. D., on sterility.......... 159
Delirium tremens, Dr. Stokes on .... 457
Delivery, without consciousness .... 132
Development of the embryo ........ 508
Diarrhœa, Dr. Copland on ........ 47
Dickson, Dr. case of ascites ........ 177
Digitalis, use of in some affections of the head .................... 57
Diphlogenesis, monstrosity by ...... 231
Discharge, mucous, from the uterus.. 222
Discharge of a fœtus from umbilicus, 162
Dislocations of the astragalus ...... 520
Disorders, mental, Dr. Uwins on .... 49
Dropsical ovaria, spontaneous cures of 206
Dropsy of the uterus, case of ...... 117
Dropsy, use of iodine in............ 259
Duodenum, diseases of, Dr. Bright on, 1
Dupuytren on hydatid tumors of wrist, 241
Dupuytren on club-feet............ 242
Dupuytren on encysted tumors...... 242
Dupuytren on syphilis & syphiloid diseases ...................... 243
Dura mater, fungoid tumour of the .. 526
Dyspepsia, Dr. Stokes on .......... 459
Dysphagia, from aneurism of the aorta 261

E.

Ear, inflammation of, in the insane .. 201
Easton, Mr. on spontaneous cholera.. 158
Easton, Mr. on dysentery .......... 159
Easton, Mr. on hæmatemesis........ 159
Easton, Mr. on ext. use of croton oil.. 159
Eclampsia in infants .............. 240
Edinburgh University, causes of its decline ...................... 315
Elliotson, Dr. on glanders in man.... 10
Embryo in mammiferous animals.... 508
Encephaloid disease of the lung...... 439
Encephaloid disease of the kidney.... 517
Encysted tumors of the liver........ 11
Encysted tumors of the kidney ...... 23
Encysted tumor on the larynx ...... 519
Enlargement, enormous, of the uterus 200
Epizootics ...................... 506
Essays, Surgical, by Mr. B. Cooper .. 137
Establishments, quarantine ........ 234
Excoriation-sore, Mr. Johnson on the 539
Extra-uterine peritoneal pregnancy .. 239
Eye, diseases of, Mr. Lawrence on.... 40
Eyes, malformations of the ........ 370
Eyes, errors in number ............ 373
Eyes, errors in position ............ 374
Eyes, errors in size................ 375

F.

Facial hemiplegia; use of phosph. ext. 204
Factory commission, report of the .. 390
Factory commission, organization of.. 391
Factory commission, its instructions.. 392
Factory commission, its mode of inquiry ...................... 393
Factory report, Sir David Barry's.... 393
Factory report, Dr. Loudon's........ 398
Factory report, Dr. Hawkins' ...... 400
Factory report, Dr. Woolriche's .... 401
False conceptions, Dr. Montgomery on 124
Fatty discharge from the bowels .... 8

Fecula, adulterations of............ 516
Fecundity, causes of .............. 160
Fecundity greater in the country .... 162
Female jurors, ignorance of ........ 122
Fibrinous polypi of the heart ...... 551
Fistula ani, Graefe's treatment of. .. 219
Fistula, congenital, in the neck, memoir on ...................... 481
Flatfootedness, treatment of ........ 520
Fletcher, Dr. his case of apoplectiform disease........................ 164
Flexure, abnormal, of the colon .... 200
Fœtal heart, sounds of ............ 105
Fœtus, discharge of a, from umbilicus 162
Fœtus breathing in utero .......... 163
Fœtus, turning of, by the head...... 237
Foreign medical politics............ 561
Fracture of the lower jaw, Dr. Chester on .......................... 469
Fractured cervix femoris, bony union, 170
Fractured limbs treated by plaster-moulds ...................... 492
Franklin, Sir William—his death.... 572
Fungating venereal sore, Dr. Hart on, 165
Fungoid tumor of the dura mater.... 526

G.

Galvanism, misapplication of, in physiological experiments ............ 226
Gangrene, spreading, amputation in.. 265
Gattei's new lithotome ............ 203
George, Mr. on the small-pox ...... 59
German Whey-Establishments...... 197
Glanders in the human subject...... 10
Glasgow, diseases of .............. 158
Gluteal artery, wound and ligature of, 165
Goethe, last illness of.............. 499
Gonorrhœa, cases of, by Mr. Johnson, 269
Gonorrhœa, simple treatment of .... 269
Graefe, Baron, his treatment of congenital omphalocele .............. 221
Graefe, Baron, his surgical report.... 218
Graefe, Baron, his new ligature instrument ........................ 218
Graefe, Baron, his treatment of polypi 219
Graefe, Baron, his treatment of fistula ani .......................... 219
Grant, Dr. his introductory lecture .. 152
Graves, Dr. on the secretion of air .. 442
Grossesse apparente, ou fausse...... 114
Guthrie, Mr. his introductory lecture 153
Guthrie, Mr. case of ligature of the common iliac.................... 278
Guthrie, Mr. on stricture .......... 548

H.

Hæmatemesis, Dr. Cumming on .... 441

Hæmorrhage, uterine, case of ...... 156

Hæmorrhage, secondary, case of .... 250

Hæmorrhage, secale cornutum in.... 451

Hart, Dr. on the fungating venereal ulcer ........................ 167

Hawkins, Mr. on encysted tumors of the liver ...................... 11

Hawkins, Mr on hydatidic tumors.. 19

Hawkins', Mr. cases of diseased testis, 247

Head, bloody tumors of the ........ 210

Hearing through apertures from the trephine ...................... 516

Heart, fœtal, sounds of........ .... 101

Heart, sounds of, explained by Laennec, Turner, Corrigan, Williams, Pigeaux, Majendie, Rouanet, Carlile, and Hope............ .... 133 & 134

Heart, differences in the fœtal & adult 163

Heart, presence of a needle in the.... 235

Heart, symptomatic affections of the.. 494

Heart, fibrinous polypi in cavities of.. 551

Hermaphrodism, M. Castel on ...... 236

Hernia, strangulated, Mr. Key on ... 80

Hernia, umbilical, of the gravid uterus 164

Hernia, femoral, thickness of sac in.. 204

Hetling, Mr. his introductory lecture, 181

Hope, Dr. on the intestinal glands in cholera ............ .......... 67

Hope's, Dr. illustrations of morbid anatomy ...................... 296

Hydatid tumors of the wrist........ 241

Hydatidic disease of the testicle ..... 245

Hydrometra, case of .............. 117

Hyosciamus, seeds of, poisoning from, 205

Hypertrophy of the mamma, cases of 464

Humours of the eye, nutrition, &c. of 505

I.

Idiopathic phlebitis, case of ........ 253

Iliac, common artery, ligature of.... 278

Infant, amputation in an .......... 191

Infants, eclampsia in.............. 240

Inflammation, Mr. Rogerson on .... 320

Inflammation, a general cause of disease ........................ 321

Inflammation, divisions of.......... 322

Inflammation, Dr. Phillips on ...... 324

Inflammation, theory of............ 324

Influenza, Dr. Ward on the ........ 364

Inhalation of iodine, &c. in phthisis.. 420

Injuries of the head, Mr. B. Cooper on 556

Insane, otitis in the .............. 201

Insanity, curability of, Dr. Uwins on.. 56

Institute, French, proceedings of .... 231

Intestinal glands, state of, in cholera.. 67

Intestinal tube, state of, in cholera .. 76

Intestine, wounded, cases of........ 182

Intestines, wounds of, B. Cooper on, 141

Intoxication, asphyxia during ...... 196

Iodine against salivation .......... 205

Iodine in pseudo-syphilitic affections, 211

Iodine used in cases of tumor ...... 251

Iodine used in dropsy.............. 258

Iodine to check salivation .......... 455

Iodine, remarks on its effects........ 467

Irritability, nervous, utility of digitalis 58

Irving, Mr. his character by Dr. Uwins 55

J.

Jaundice and cerebral affection.. 454, 479

Jaw, on fracture of the lower ...... 469

Jejunum, rupture of the.............. 143
Johnson, Mr. cases of gonorrhœa.... 269
Johnson, Dr. on the intimate union of medicine and surgery............ 282
Johnson, Dr. on the necessity of having one faculty of medicine ...... 284
Johnson, Mr. H. J. on venereal sores 529
Joints, ulceration of, Mr. Key on .... 25
Jurisprudence, medical ............. 120
Jurors, female, ignorance of........ 122

K.

Kennedy, Dr. on obstetric auscultation 92
Key, Mr. on ulceration of the joints.. 25
Key's, Mr. plan of operating in hernia 80
Key, Mr. on dangers of dividing the sac 81
Kidney, aqueous encysted tumor of.. 23
Kidney, encephaloid disease of the .. 517
Kiernan, Mr. on anatomy of the liver 305

L.

Labour, premature, induction of .... 503
Labour impeded by malformations of the fœtus...................... 489
Lacerated perinæum, operation for .. 510
Laennec on the sounds of the heart.. 133
Lancaster Lunatic Asylum........... 546
Langford, on the Asiatic cholera ... 145
Larynx, encysted tumour on the .... 519
Law, interpretation of, in cases of pregnancy ...................... 121
Lawrence, Mr. on diseases of the eye.. 40
Lawrence, Mr. on purulent ophthalmia 43
Lawrence, Mr. on strumous ophthalmia 44
Lectures, introductory, by Drs. Grant and Watson, and Messrs. Wardrop and Guthrie ................... 152
Lee, Mr. E. on nervous affections.... 148
Leucorrhœa, Boivin and Duges on .. 222
Leucorrhœa, secale cornutum in .... 451
Life continuing after craniotomy .... 204
Ligature instrument, Baron Graefe's, 218
Ligature, amputation of the leg by a.. 235
Lithotome, new, by Gattei ......... 203
Lithotrity, M. d'Etiolles on ........ 232
Liver, aqueous encysted tumors of the 11
Liver, Mr. Kiernan's anatomy of the 305
Liver, lobules of the ............... 307
Liver, portal canals of the.......... 309
Liver—hepatic veins, &c. ........... 311
Liver, structure of its lobules ...... 312
Liver, red and yellow substances of the 313
Liver, Mr. Geddes on abscess of the.. 365
Liver, inflammation of the ........ 369
Liverpool Medical Society......... 188
Lock Hospital, report from ........ 269
Louis XIV., birth of .............. 159
Lumbago, with metastasis to testicle.. 544
Lung, encephaloid disease of the .... 439
Lung, calcareous incrustation on the.. 501
Lungs, diseases of, Dr. Williams on.. 132

M.

Macilwain, Mr. on porrigo.......... 348
Macilwain, Mr. on cure of nævi .... 174
Mackintosh, Dr. on amenorrhœa .... 161
Majendie on the sounds of the heart.. 133
Males and females, numbers of...... 508
Malformations of the œsophagus .... 230
Malformation of the eyes, Dr. Seiler on ............................ 370
Malformations of fœtus impeding labour ......................... 489
Mamma, cases of hypertrophy of .... 464
Mammæ, sympathy between them and the uterus ..................... 171
Mammary abscess, Dr. Beatty on.... 448
Marshall, Mr. case of ovarian tumor, 177
Materia medica, Dr. Thompson's .... 403
Maxwell, Dr. on uterine hæmorrhage, 156
Medical jurisprudence .............. 120
Medical politics—Aldersgate question 185
Medical and Physical Journal, death of 190
Medical reform, remarks on ........ 278
Medical topography of Gowhattee.... 363
Medical problems, by Dr. Griffin .... 454
Medical statistics and reform......... 567
Medical reform .................... 560
Middlemore, Mr. on ulceration of the eyelid .......................... 461
Mind, disorders of, Dr. Uwins on.... 49
Monstrosity by diphlogenesis......... 231
Montgomery, Dr. on pregnancy ..... 92
Montgomery on moles ............ 124
Montgomery on the corpora lutea.... 127
Morbid anatomy, Dr. Hope on ...... 66
Morbid anatomy, Dr. Carswell on.... 66
Morbid anatomy, Cruveilhier on .... 66
Mortality in armies................. 231
Mortality in different countries...... 234
Mucous discharge from the uterus .. 222
Muller's experiments upon the blood 501
Muller's experiments on spinal nerves 225
Muscles, nervous affections of the.... 148

N.

Nævi materni, Graefe's treatment of.. 219
Nævi, treatment of, by seton........ 174
Naval surgeons, rules affecting....... 188
Necrosis of the thigh, case of ...... 250
Nerves of sense, paralysis of the...... 213
Nerves, spinal, Muller's experiments 225
Nerves, Mr. Swan on the anatomy of 334
Nerves, Mr. Swan on the diseases of.. 423
Nerves, wounds of the ............ 423
Nerves, excision of parts of ........ 424
Nerves, tumors in ................ 431
Nervous affections of the muscles.... 148
Neuralgic affection of stumps ....... 555
Nitrate of silver in cynanche laryngea 155
Nitric acid, effects of a large dose of.. 64
Numann's experiments on small-pox 208

O.

Obstetric auscultation, Dr. Kennedy 92

Œsophagotomy, Mr. Arnott's case of 172

Œsophagus, malformations of the .. 230

Œsophagus, rupture of the ........ 230

Œsophagus, dilatation of the ...... 230

Operation, Cæsarian, case of........ 207
Operation, Cæsarian, case of........ 221
Ophthalmia, purulent, Mr. Lawrence 43
Ophthalmia, strumous ............ 44
Orbit, wound of the .............. 253
Ore, Mr. his case of extraordinary pulmonary abscess ................ 192
Osteology, Dr. Witt on ............ 149
Otic ganglion, Arnold's ............ 498
Otitis, external, in the insane........ 201
Ova in mammiferous animals ...... 509
Ovaria, dropsical, spontaneous cures of 206
Ovarian tumor, case of ............ 177

P.

Paine, Dr. on cerebral diseases...... 433
Palsy, facial, use of phosphorus in .. 204
Pancreas, diseases of, Dr. Bright on.. 1
Paracentesis abdominis, case of .... 177
Paralysis of the nerves of seeing, hearing, and smelling .............. 213
Par vagum, Mr. Swan's experiments on 425
Par vagum, supposed inflammation of 428
Par vagum, pressure on the ........ 430
Paterson, Dr. on amenorrhœa ...... 171
Pathological anatomy, works on .... 289
Paul, Dr. case of amputation in an infant ........................ 191
Percussion of the lungs............ 339
Percussion of the heart ............ 343
Percussion of the abdomen ........ 345
Percussion of the liver ............ 346
Pericardiac effusion, extraordinary quantity of .................... 178
Pericarditis induced by a needle in the heart ........................ 235
Perinæum, lacerated, operation for .. 510
Philadelphia, cholera gazette of...... 178
Phillips, Dr. on inflammation ...... 324
Phlebitis, case of idiopathic ....... 253
Phlegmonous erysipelas, remarks on.. 323
Phosphorus used extensively in palsy, 204
Phthisis, pulmonary, use of whey in.. 197
Phthisis, Dr. Stokes on ............ 257
Physic and surgery, intimate union of 282
Physiology of the fœtal heart ...... 163
Pigeaux on the sounds of the heart .. 133
Piorry, M. on mediate auscultation .. 337
Placentary bruit, Dr. Kennedy on.... 101
Plaster-moulds for fractured limb.... 492
Pleuritis, Dr. Stokes on ............ 257
Plica polonica, M. Boismont on .... 215
Poisoning from hyosciamus seeds .... 205
Poisons, Dr. Roupell on............ 62
Politics, medical.................. 561
Polypi, treatment of, by Graefe...... 219
Porrigo, Mr. Macilwain on ....... 348
Porrigo, characters and origin of .... 350
Porrigo, contagiousness of.......... 351
Porrigo, dietetic management of .... 354
Porrigo, medicinal treatment of .... 358
Porrigo decalvans treated by tartar emetic ..... .................. 453
Porter, Mr. on aneurism of the aorta 261
Porter, Mr. on amputation in spreading gangrene .................. 265
Portrait of Joshua Brookes ........ 189
Preceptor, the...................... 151
Pregnancy, visible signs of.......... 96
Pregnancy, tangible signs of........ 98
Pregnancy, audible signs of ........ 100
Pregnancy, simulated.............. 119
Pregnancy, compound ............ 112
Pregnancy, complicated............ 113
Pregnancy, peritoneal, extra-uterine.. 239
Progress of physiological science .... 493
Provincial schools of medicine ...... 180
Prussian divisions of medical men .. 563
Prussian armies, revaccination of.... 195
Pseudo-pregnancy ................ 114
Pseudo-syphilitic affections of the throat ........................ 211
Pulmonary abscess, extraordinary case 192
Pulmonary diseases, acetas plumbi in 193
Pulse, fœtal, frequency of the ...... 105
Purification of dissecting theatres.... 516
Pyrmont, vapour cave at .......... 201

Q. and R.

Quarantine establishments, inutility of 234
Reform meeting in Ireland ........ 571
Reform, medical, remarks on........ 278
Reform, medical.................. 560
Regulations, new, of Navy Med. Board 188
Respiration of fœtus in utero ...... 163
Retroversio uteri, case of .......... 156
Revaccination of the Prussian armies 195
Rheumatism, M. Bouillaud on ...... 254
Rice, the cause of cholera ! !........ 180
Robertson, Mr. on cynanche laryngea 155
Roche, M. on alterations of the blood 381
Rogerson, Mr. on inflammation .... 320
Rot of sheep communicated to man.. 484
Rouanet on the sounds of the heart.. 134
Roupell, Dr. on poisons............ 62
Rupture of the jejunum............ 142
Rupture of the spleen.............. 144

S.

Sac, thick, in femoral hernia........ 204
Salicine for ague.................. 482
Salivation arrested by iodine........ 205
Scudamore, Sir Charles, on iodine .. 420
Secale cornutum, Dr. Negri on...... 450
Secale cornutum, Dr. Beatty on the.. 438
Secondary symptoms, cases of ...... 537
Secondary hæmorrhage, case of...... 250
Seiler, Dr. on malformation of the eyes 370
Sense, nerves of, paralysis of........ 213
Seton, use of, in nævi ............ 174
Signs of pregnancy................ 96
Silver, nitrate of, in cynanche laryngea 155
Simpton, Mr. on clots in the bladder.. 183
Simulated pregnancy.............. 119
Sinapisms to the mammæ, in amenorrhœa ........................ 171
Skeletons, best mode of preparing.... 150

Small-pox, Mr. George on.......... 59
Sonderland's experiments on cow-pox repeated .................... 208
Sounds of heart variously explained .. 133
Spina ventosa .................... 528
Spinal nerves, Muller's experiments on 225
Spleen, rupture of ................ 144
Statistics, medical ................ 234
Stanley, Mr. on osseous union of a fractured cervix femoris.......... 170
Sterility, curious cases of .......... 159
Still-born children, auscultation applied to ...................... 164
Stokes, Dr. on phthisis ............ 257
Stokes, Dr. on pleuritis biliosa ...... 257
Stokes, Dr. on dysphagia .......... 257
Stokes, Dr. on diptherite .......... 258
Stokes, Dr. on laryngeal phthisis .... 258
Stokes, Dr. on dropsy.............. 259
Stokes, Dr. on sympathy .......... 259
Stokes, Dr. on delirium tremens, &c. 457
Stomach-pump, new application of.. 183
Strangulated hernia, Mr. Key on.... 80
Stricture, Mr. Guthrie on.......... 548
Stumps, neuralgic affection of ...... 555
Sulphate of magnesia made agreeable! 438
Surgery and physic, intimate union of 282
Surgeons, shades of, in Prussia...... 564
Surgical Essays by Mr. B. Cooper.... 137
Swan, Mr. his plates of the nerves .. 334
Swan, Mr. on diseases of the nerves.. 423
Symptomatic affections of the heart.. 494
Syphilis and syphiloid diseases ...... 242

T.

Tangible signs of pregnancy ........ 98
Tarentism, account of.............. 239
Tartar-emetic in croup ............ 212
Tartar emetic solution for porrigo .. 453
Tartarized antimony in cholera...... 145
Teeth, as the nuclei of urinary calculi 475
Testicle, hydatidic, disease of........ 245
Testis, diseased, cases of............ 247
Thompson, Dr. his elements of materia medica ...................... 403
Throat, diseases of, iodine in........ 211
Thymus gland, development of the .. 487
Tooth lodged in the bronchus ...... 471
Torsion of the arteries, performed by Clot Bey ...................... 491
Tracheotomy, successful case of .... 473
Transactions of the Calcutta Society.. 361
Traumatic tetanus, case of ........ 555
Tumor ovarian, case of ............ 177
Tumor, cases of, treated by iodine.... 251
Tumors, bloody, of the head........ 210
Tumors, hydatid, of the wrist ...... 241
Tumors, encysted, of the head ...... 242
Turnbull, Dr. on veratria .......... 405
Turner, Mr. on the sounds of the heart 133
Turning of the fœtus by the head.... 237
Turpentine, injurious effects of, in gonorrhœa .................... 270
Typhus, M. Bouillaud on .......... 255
Typhus fever, M. Bouillaud on...... 514
Tytler, Dr. doctrine of cholera ...... 180

U.

Ulceration of the eyelid, Mr. Middlemore on ...................... 461
Ulcerative process in joints ........ 25
Umbilicus, discharge of a fœtus from 162
University of Edinburgh, causes of its decline ...................... 315
Urinary calculi, teeth the nuclei of .. 475
Uterine hæmorrhage, prevention of.. 446
Uterus, dropsical, case of .......... 117
Uterus, hæmorrhage from.......... 156
Uterus, retroversion of ............ 156
Uterus, respiration of fœtus in the .. 163
Uterus, gravid, hernia of .......... 164
Uterus, enormous enlargement of.... 200
Uterus, mucous discharges from .... 222
Uwins, Dr. on mental disorders .... 49
Uwins, Dr. on the character of the Rev. Edw. Irving .............. 55
Uwins, Dr. on the curability of insanity 56
Uwins, Dr. on asthenic disease of the brain ...................... 57
Uwins, Dr. on nervous irritability.... 57

V.

Vaccine, Traité de la, par M. Bousquet 417
Vapour cave at Pyrmont .......... 201
Venereal ulcers, Mr. Johnson on .... 529
Venereal ulcers, cases of .......... 533
Venereal condylomata, lotion for .... 204
Venereal disease, antiquity of the.... 331
Veratria, Dr. Turnbull on.......... 405
Veratria, mode of using............ 406
Veratria in affections of the heart.... 407
Veratria in neuralgic affections...... 411
Veratria in rheumatism............ 413
Veratria in paralysis .............. 413
Veratria in dropsy................ 414
Vermination, Dr. Alexander on .... 449
Visible signs of pregnancy.......... 96

W.

Wardrop, Mr. on effects of bleeding.. 476

Wardrop, Mr. his introductory lecture 153

Warts, venereal, lotion for.......... 204

Watson, Dr. his introductory lecture 152

Westminster Medical Society ...... 187

Whey-establishments in Germany .. 197

Williams, Dr. on auscultation ...... 132

Williams, Dr. on plessimeters ...... 132

Williams, Dr. on sounds of the heart 133

Witt, Dr. his osteology ............ 149

Women, fecundity of, in different climates ...................... 160

Wound of the gluteal artery, case of 165

Wound of the intestine, case of...... 182

Wound of the orbit, case of ........ 253

Wounds of the abdomen, B. Cooper on 391

Wrist, hydatid tumors of the........ 241

Writ, 'de ventre inspiciendo' ...... 123

